# Biodegradation of Di (2-Ethylhexyl) Phthalate by a novel *Enterobacter* spp. Strain YC-IL1 Isolated from Polluted Soil, Mila, Algeria

**DOI:** 10.3390/ijerph17207501

**Published:** 2020-10-15

**Authors:** Imane Lamraoui, Adel Eltoukhy, Junhuan Wang, Messaouda Lamraoui, Amer Ahmed, Yang Jia, Tiegang Lu, Yanchun Yan

**Affiliations:** 1 Biotechnology Research Institute, Chinese Academy of Agricultural Sciences, Beijing 100081, China; imane.lamraoui1@gmail.com (I.L.); lutiegang@caas.cn (T.L.); 2 Botany and Microbiology Department, Faculty of Science, AL-Azhar University, Assiut 71524, Egypt; adelaly@azhar.edu.eg; 3 Graduate School of Chinese Academy of Agricultural Sciences, Beijing 100081, China; 82101171004@caas.cn (J.W.); jia_yang@outlook.com (Y.J.); 4 Department of Microbiology, Faculty of Nature Science and Life, University of Bejaia, Bejaia 0600, Algeria; lamraoui.messaouda@gmail.com; 5 Department of Life Sciences, University of Siena, 53100 Siena, Italy; aa.biotechiub@gmail.com

**Keywords:** Di (2-Ethylhexyl) phthalate, biodegradation, *Enterobacter* spp., endocrine disruptor, degradation pathway, bioremediation

## Abstract

Di-(2-ethylhexyl) phthalate (DEHP) is one of the phthalic acid ester representatives and is mainly used as a plasticizer to endow polyvinyl chloride plastics with desirable physical properties. It is synthesized in massive amounts worldwide. Many studies have proved the adverse effects of DEHP on human health and wildlife. DEHP is labeled as an endocrine disruptor which causes human reproductive problems. *Enterobacter* spp. YC-IL1, a novel isolated strain from contaminated soil, was identified by 16S rRNA gene analysis and electronic microscope. It is capable of efficiently degrading DEHP (100%) and a wide range of phthalic acid ester PAEs, particularly those containing side chains with branches, or ring structures such as dutylbenzyl phthalate and dicyclohexyl phthalate, which are hard to degrade, with, respectively, 81.15% and 50.69% degradation after 7 days incubation. YC-IL1 is an acido-tolerant strain which remained in pH values lower than pH 5.0 with the optimum pH 7.0 and temperature 30 °C. The DEHP metabolites were detected using HPLC-QQQ and then the degradation pathway was tentatively proposed. Strain YC-IL1 showed high DEHP degradation rate in artificially contaminated soil with 86% removed in 6 days. These results indicate the application potential of YC-IL1 in bioremediation of PAE-polluted sites, even the acidic ones.

## 1. Introduction

Phthalic acid esters (PAEs) are a large class of organic molecules extensively used in plastic and packing product industries. This class includes di-methyl phthalate (DMP), di-ethyl phthalate (DEP), di-n-butyl phthalate (DBP), di-n-nonyl phthalate (DNP), di-n-decyl phthalate (DDP), butylbenzyl phthalate (BBP), di-n-propyl phthalate (DPrP), di-n-butyl phthalate (DBP), and di-2-ethyl hexyl phthalate (DEHP), among others [1]. DEHP, also called bis(2-ethylhexyl) phthalate, is the most extensively used member of PAEs in the plastic industry. DEHP is made up of two eight-carbon chains (ethylhexene) linked to phthalic acid via two ester bonds giving rise to a compound with the chemical formula C_24_H_38_O_4_ and molecular weight of 390.56 g/mol. DEHP is lipophilic liquid soluble in organic solvents but poorly soluble in water and lacking both color and odor [2]. It is synthesized in massive amounts, with its global production surpassing 4 million tons annually. It is used as a softener in polyvinyl chloride plastics (PVC) to impart them with the required flexibility and durability [3]. Hence, DEHP is present in many products, especially medical devices such as hospital tubing and blood storage bags [4], and a wide variety of consumer products such as cosmetics, food wrapping, wire and cable insulation, automobile parts, furniture materials, and toys and infant products. The percentage of DEHP in different products varies from 10–60% [5,6].

Like PAEs, DEHP is not covalently linked to PVC and therefore it can easily leach from DEHP-containing materials into the environment in the manufacturing, consumption, and disposition processes [7,8,9]. Hence, DEHP is widely distributed in the environment. For instance, DEHP has been detected at varying concentrations in different environments such as freshwater, sediment, soils, particulate matter, landfill leachate, and sewage sludge [10,11,12,13], and in ambient air samples in trace levels. The leakage rate of DEHP into the environment is affected by many factors, including temperature, pH, organic particles, and solvents [14]. It is not therefore surprising that DEHP is found inside living organisms including human beings. In fact, measurable amounts of DEHP can be detected in different body organs such as the liver, kidney, and testes and body fluids such serum, urine, and milk [15,16]. Epidemiological studies have also correlated DEHP contamination to various pathological conditions such as cancer, diabetes, allergies, and asthma [16]. Notably, DEHP has been classified as an endocrine-disrupting chemical (EDC) that interrupts the normal function of the endocrine system and adversely affects human development, reproduction, and neurological, respiratory, and immune functions [17]. Furthermore, repeated DEHP exposure may cause DNA damage, apoptosis, and cell proliferation [18]. Human exposure to DEHP may occur through inhalation, ingestion, or dermal adsorption and subsequent absorption [19,20]. In fact, agriculture manipulation with plasticized PVC instruments can lead to the accumulation of DEHP in the soil which, in turn, is absorbed by plants and enters into the food chain cycle [21]. In this scenario, DEHP has been listed as a priority hazardous substance by the European Community, the United States Environmental Protection Agency, and the China National Environmental Monitoring Center [12,22,23]. Hence, removal of DEHP from the environment is of great importance to limit its effect on human health and ecological balance.

Different methods have been adopted to eliminate DEHP, along with other PAEs, from the environment, such as hydrolysis [24], photo-degradation [25], pulse radiolysis and electron beam radiolysis [26], adsorption [27], subcritical water extraction [28], and microbial degradation. The latter is the most plausible and favorite method due to its safety, high efficiency, and low cost when compared with the other methods. In addition, microorganisms are versatile, adaptive, and ecosystem friendly [29]. In this scenario, numerous bacterial genera with the capability of degrading DEHP have been isolated, such as *Rhodococcus* [30], *Gordonia* [31], *Agromyces* [32], *Pseudomonas* [33], *Sphingomonas* [34], *Arthrobacter* [35], *Achromobacter* [36], *Bacillus* [19], *Providencia* [37], *Acinetobacter* [38], and *Mycrobacterium* [39].

To our knowledge, the present work is the first report on a novel bacterium identified as *Enterobacter* spp. YC-IL1 that can degrade DEHP and a wide range of PAEs. It was isolated from soil samples from a plastic waste collection area in Mila province, Algeria. This novel strain showed high degradation efficiency toward DEHP and several PAE pollutants. The effects of environmental factors such as pH, temperature, and salinity were also assessed. Broad substrate spectrum was tested and cultures collected from different time periods were analyzed by HPLC-QQQ to identify DEHP intermediates during the metabolic process, and a potential metabolic pathway for DEHP catabolism was deduced. The maximum and minimum DEHP concentrations that can be degraded effectively were also measured to determine the range of applicability of this novel strain. Finally, we examined bioremediation potential of this novel strain on DEHP-contaminated soil.

## 2. Materials and Methods

### 2.1. Chemicals and Media

All PAEs used in this study were purchased from Sinopharm Chemical Reagent Beijing Co., Ltd., China and were of analytical grade with purities above 99.0%. They were dissolved in methanol (HPLC grade), sterilized by membrane filtration (0.22 μm), and diluted appropriately to the required concentrations before use. The PAEs employed in the present study include DMP, DEP, DPrP, DBP, DNP, DDP, BBP, DEHP, phthalic acid bis-n-pentyl ester (DAP), dicyclohexyl phthalate (DCHP), di-n-hexyl phthalate (DHP), mono-ethylhexyl phthalate (MEHP), di-n-heptyl phthalate (DHpP) and phthalic acid (PA). Other chemicals were of analytical grade and solvents also were of HPLC grade.

Two different media were used, namely Trace Element Medium (TEM) and Luria-Bertani (LB) medium for enrichment and isolation, respectively. The TEM consists of the following chemicals in (g/L distilled water): (NH4)_2_SO_4_ 2.0, Na_2_HPO 1.5, K_2_HPO_4_ 1.5, MgSO_4_ 0.2, CaCl_2_ 0.01, and agar 15 (for only solidified media), and 100 μL stock trace element solution (TES). TES is, in turn, comprised of (in g/L): FeSO_4_ 50, ZnSO_4_ 2.2, CuSO4 0.3, MnSO_4_ 14.3, CoSO_4_ 1.2, Na_2_MoO_4_ 0.2, and Na_2_WO_4_ 2.3. LB medium contains in (g/L): peptone 10, yeast extract 5, NaCl 10, and agar 15 [40]. The pH values were adjusted appropriately using NaOH or HCl (2 M) before autoclaving at 121 °C for 20 min.

### 2.2. Instrumental Analysis

Bacterial growth was determined by measurement of the optical density (OD) at 600 nm using a UV-VIS Spectrophotometer (Thermo Scientific, Waltham, MA, USA). PAE concentrations were detected by GC-2010 system (SHIMADZU, Kyoto, Japan) equipped with a HP-5 capillary column (in radium 0.25 mm, length 30 m, membrane thickness 0.25 μm) and a flame ionization detector (FID) at 300 °C, with an injection volume of 5 μL. Ultrahigh purity nitrogen served as the carrier gas at a flow rate of 1.51 mL/min. The column temperature gradually increased from 160 to reach 280 °C at the rate of 10 °C/min and then was held for 4 min at 280 °C, carrier gas for FID-H_2_ (40 mL/min) and air (400 mL/min). Samples to be analyzed were extracted with the equal volume of hexane and filtered through 0.22 μm membrane (ANPEL, Shanghai, China). The metabolites of DEHP were analyzed using liquid chromatogram (LC, Agilent 1260) coupled with a triple quadrupole mass spectrometer (QQQ, Agilent G6400).

### 2.3. Enrichment and Isolation of DEHP-Degrading Strain

Soil samples used in the present study were collected from Mila province, Algeria. A total of 5 mg of soil was added to 20 mL TEM supplemented with 100 mg/L DEHP and incubated in a rotary shaker (180 rpm) at 30 °C for 7 days. After incubation, 1 mL of the enriched culture was transferred to 20 mL fresh TEM supplemented with 200 mg/L DEHP. This enrichment process was repeated with 100 mg/L DEHP increment until the final concentration reached 400 mg/L. After 28 days of enrichment and domestication, an aliquot of the resulting culture were spread on a TEM plate supplemented with 100 mg/L of DEHP as sole carbon source and further incubated at 30 °C. Single colonies of different sizes, morphology and colors were purified in LB plates. The degradation capability of purified isolates were tested by incubation in 20 mL TEM supplemented with 100 mg/L DEHP for 7 days, and DEHP concentration was detected by GC. PAEs were detected by GC and the chromatographic response (peak area) was linearly correlated with the concentration of PAEs. Hence, the PAE degradation rate was calculated by the change of chromatographic signal compared to control. In other words, degradation rate = (peak area of control-peak area of sample)/peak area of control. Uncultured TEM supplemented with PAEs was used as control.

### 2.4. Molecular Identification of DEHP-Degrading Strain

The isolated strain was identified through 16S ribosomal RNA (rRNA) gene analysis as previously described [31]. Briefly, genomic DNA was extracted using Bacterial Genomic DNA Extraction kit (Takara, Ostu, Japan) and 16S rRNA gene sequence was amplified using two universal primers: 27 F (5′-AGAGTTTGATCCTGGCTCAG-3′) and 1492 R (5′-GGTTACCTTGTTACGACTT-3′) [31]. The PCR products were run through a gel electrophoresis for 30 min then sequenced by Sangon Biotech Co., Ltd., Shanghai, China. The obtained sequence was deposited into GenBank under the accession number MT002728 and searched against the Basic Local Alignment Tool (BLAST) to visualize species with the highest similarity to our newly identified strain. A phylogenetic tree was constructed using neighbor-joining algorithm and MEGA 6.0 software (MEGA Software Development Team directed by Sudhir Kumar, Phoenix, AZ, USA)

### 2.5. Inoculum Preparation

To prepare the inoculum, a pure culture of strain YC-IL1 obtained from individual colonies was inoculated into LB and incubated at 30 °C in a rotary shaker at 180 rpm. After 24 h cultivation, cultures were harvested by centrifugation at 4500 rpm for 5 min, washed twice with fresh TEM, and re-suspended in fresh TEM to set a cell density of absorbance at 600 nm (A_600_) to 0.8. One percent of this suspension (approximately 1.0 × 10^8^ cells/mL) was used as inoculum for PAEs biodegradation following studies.

### 2.6. Effects of Environmental Factors on DEHP Degradation

In this series of experiments, the degradation efficiency of YC-IL1 toward DEHP (100 mg/L), was assessed under different physiochemical factors such as temperature (15, 20, 25, 30, 35, 40, 45 °C), pH (2, 3, 4, 5, 6, 7, 8, 9, 10, 11) and salinity (0, 0.5, 1, 1.5, 2, 4% NaCl). All samples were incubated for 7 days and remaining DEHP concentration was detected by GC. All experiments were performed in triplicate and uncultured TEM supplemented with DEHP (100 mg/L) was used as control.

### 2.7. Degradation Efficiency of DEHP at Maximum and Minimum Concentrations

In this series of experiments, the degradation efficiency of YC-IL-1 was assessed using different concentrations of DEHP (lower concentration (1, 2, 5, and 10 mg/L) or higher concentration (50, 100, 200, 300, 400, 500 and 1000 mg/L)) in order to determine the minimum and maximum concentration of DEHP that can be degraded by YC-IL-1. After incubation for 7 days, the DEHP concentration was analyzed using GC and the degradation rate was calculated. All experiments were performed in triplicate and uncultured TEM supplemented with corresponding DEHP concentration was used as control.

### 2.8. Substrate Utilization Tests

In this series of experiments, the capability of strain YC-IL1 to degrade other types of PAEs was assessed. Briefly, the strain YC-IL1 was inoculated into 10 mL of TEM medium in a 100 mL Erlenmeyer flask containing 100 mg/L of DMP, DEP, DPrP, DBP, DAP, DHP, DHpP, DOP, DNP, DDP, BBP, DEHP, DCHP, MEHP, and PA as sole carbon source. Non-inoculated cultures were served as abiotic control; all experiments were performed in triplicate. All cultures were incubated in rotary shakers (180 rpm) at 30 °C for 7 days. The residual concentrations of PAEs were determined by GC and PA concentration was measured by HPLC-QQQ (Agilent, Palo Alto, CA, USA).

### 2.9. Analysis of Intermediates

In order to explore the DEHP degradation pathway by *Enterobacter* spp. YC-IL1, intermediates of DEHP were analyzed by HPLC-QQQ. Culture samples of 1, 2, 3, 4, 5, 6, and 7 days were extracted twice using equal volume of ethyl acetate, dried by N_2_ and re-suspended in 1 mL methanol. The probable residual DEHP and its degradation intermediates produced by YC-IL1 were analyzed by HPLC–QQQ. The parameters of mass spectrometer were ESI positive (for detection of DEHP) and negative (for detection of MEHP and PA), full scan mode from m/z 50 to 800. The mobile phase was 10% water with 0.1% formic acid and 90% methanol. All data were acquired and processed using Masshunter software (Agilent, Palo Alto, CA, USA).

### 2.10. Decontamination of Artificially DEHP-Polluted Soil

In this kind of experiment, the performance of YC-IL1 to degrade DEHP in the natural environment was tested. Briefly, soil samples were collected from the top layer (0–20 cm) of a garden in Graduate School of Chinese Academy of Agriculture Science (GSCAAS) located in Beijing, Northern China. The soil was well mixed and dried at room temperature before use. The soil was passed through a sieve to remove unwanted stones, plants, and debris. This experimental soil was divided into two groups: Group (A) was treated with heat leading to its sterilization (sterilized soil), and group (B) was not heat-treated (non-sterilized soil). Subsequently, both of the two groups were artificially polluted with 100 mg/kg DEHP and inoculated with different inoculum size (0, 1, 5, 10 and 15%) of strain YC-IL1. Each inoculum size was prepared in triplicate and incubated at 30 °C for 6 days. Artificially contaminated soil without inoculation was set up as blank control. After the incubation period, DEHP residues were extracted from the soil using hexane and detected by GC.

### 2.11. Statistical Analysis

GraphPad Prism version 5.04 (GraphPad Software Inc., San Diego, CA, USA) was used to analyze the data collected from different experiments. Data are reported as mean ± SEM. Statistical analysis was performed by two-way repeated-measure ANOVA, one-way ANOVA followed by Dunnett’s post-hoc test, or two-way ANOVA followed by Bonferroni post-test using GraphPad Prism software; *p* < 0.05 was considered significant.

## 3. Results

### 3.1. Microbial Isolation and Identification of DEHP-Degrading Bacterium

**Figure 1:**
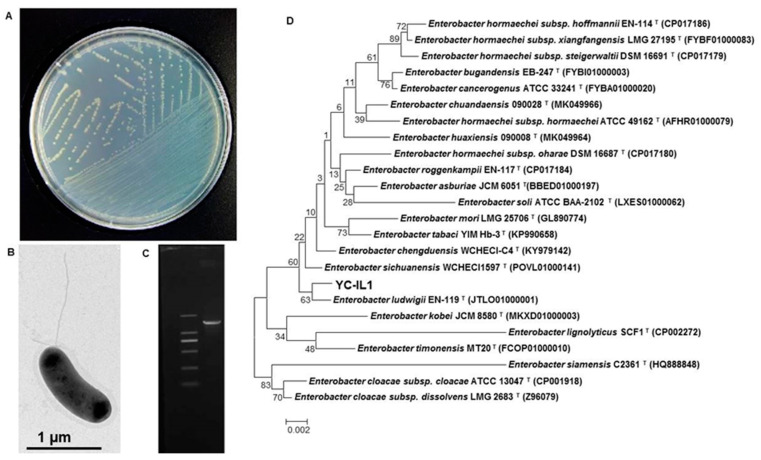
(**A**) The colonial morphology of strain YC-IL1 on TEM (Trace Element Medium) plate, (**B**) TEM investigation showing cell shape and lophotrichous flagellum, (**C**) amplified 16S rRNA gene of YC-IL1 compared with 2000 bp DNA leader, (**D**) phylogenetic tree of *Enterobacter* spp. YC-IL1 with closely related strains.

The bacterial strain YC-IL1 was isolated through a series of enrichment and domestication experiments from contaminated soil from plastic waste in Mila province, Algeria, and showed an excellent capability to degrade DEHP and a wide range of PAEs. Morphological characterization showed that YC-IL1 colonies are light yellow in TEM (Figure 1A), gram-negative, motile with flagella, and rod-shaped (Figure 1B). Phylogenetic tree analysis of the 16S rRNA gene sequence of YC-IL1 (Figure 1C) showed that it has 99.79% sequence similarity to *Enterobacter ludwigii* EN-119 (CP017279.1) and was identified as *Enterobacter* spp. YC-IL1 (Figure 1D).

### 3.2. Utilization of DEHP as Sole Source of Carbon for Growth by YC-IL1

**Figure 2:**
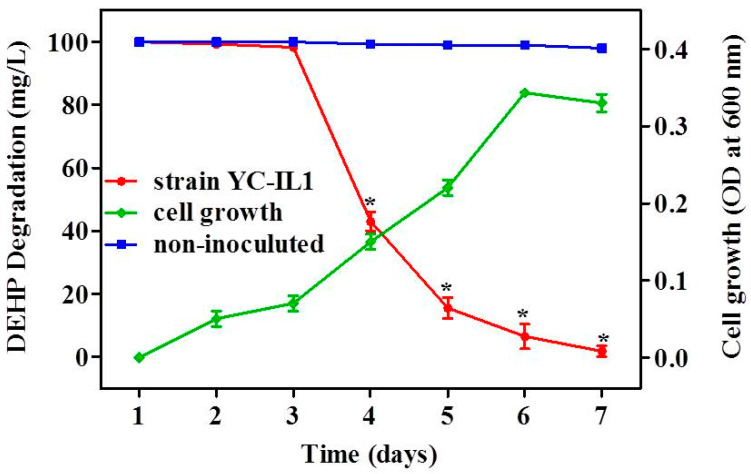
DEHP (di-2-ethyl hexyl phthalate) degradation rate and cell growth of YC-IL1 under optimized conditions. Data represent the mean ± SEM from at least three experiments. * indicates the significant difference (*p* < 0.05; two-way repeated measure ANOVA followed by Bonferroni post-test) between the media inoculated with strain YC-IL and control (non-inoculated media).

As shown in Figure 2, strain YC-IL1 grew well on TEM containing DEHP as the sole carbon source. Similarly, DEHP was significantly degraded by YC-IL1 within 7 days (100 mg/L DEHP was reduced to less than 2 mg/L after 7 days of incubation) compared to the control (non-inoculated medium; two-way ANOVA; *p* < 0.05). Anwar et al. reported that degradation of toxicants is comprised of two phases: an adaption or lag phase followed by an accelerated degradation phase [41]. Interestingly, in our studies, strain YC-IL1 showed 36 h of adaptation phase, followed by an efficient degradation phase with 57 and 84% of initial DEHP dose degraded by the fourth and fifth day, respectively.

### 3.3. Effects of Environmental Factors on DEHP Degradation

#### 3.3.1. Effect of Temperature on DEHP Degradation by YC-IL1

**Figure 3:**
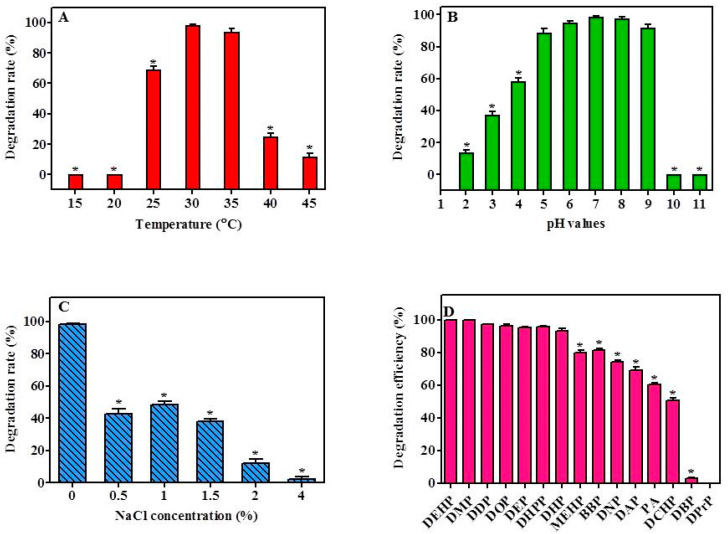
Effect of environmental factors on DEHP degradation rate by strain YC-IL1: (**A**), Effect of temperature; (**B**), effect of pH; (**C**), effect of NaCl concentration; (**D**), degradation rates of PAEs by the strain YC-IL1. The initial concentration was 100 mg/L and the degradation rate was calculated as a percentage of the transformed amount of DEHP. The degradation rate was recorded after 7 days incubation. Data represent the mean ± SEM of three independent replicates. * indicates the significant difference (*p* < 0.05; one-way ANOVA followed by Dunnett’s test) between the columns denoted with (*) and control; the controls are 30 °C for temperature effect, pH 7 for the pH effect, 0% for the NaCl concentration effect, and DEHP for PAEs degradation efficiency.

The capability of YC-IL-1 to degrade the DEHP was assessed over a wide range of temperature (15–45 °C) with 5 °C increments. As shown in Figure 3A, strain YC-IL1 was capable of efficiently degrading DEHP in a temperature range of 30–35 °C with the optimum degradation occurring at 30 °C (Figure 3A). However, at lower temperatures (15–25 °C) or higher temperatures (40–45 °C), the degradation rate was significantly decreased compared to the control (30 °C; *p* < 0.05 one-way ANOVA) probably because of low microbial growth at these temperatures. As the temperature was increased to 25 °C, the rate of degradation was remarkably increased to achieve 68.87% and the optimum degradation rate was noted at 30 °C and 35 °C with 98.40% and 94%, respectively. At higher temperatures, the degradation rate markedly decreased, recording 25% and 11.9% at 40 °C and 45 °C, respectively (Figure 3A).

#### 3.3.2. Effect of pH on DEHP Degradation by YC-IL1

As shown in Figure 3B, strain YC-IL1 efficiently degraded DEHP in a wide range of pH values (5–9) with the optimum degradation occurring at pH 7. For instance, the degradation rates obtained at pH 7, 8, and 9 were 98.4, 97.6, and 91.7%, respectively. However, decreasing or increasing pH values beyond this range resulted in a significant decline in the degradation rate. Medium with pH exceeding 10 was devoid of any DEHP degradability, whereas in medium with acidic pH, degradation rates were 37.48, 58.22, and 88.67% at pH 3, 4, and 5, respectively, suggesting that YC-IL1 is an interesting acido-tolerant strain worthy of further investigation.

#### 3.3.3. Effect of NaCl Concentration on DEHP Degradation by YC-IL1

The effect of salinity stress on the capability of YC-IL1 to degrade DEHP was assessed at different concentrations of NaCl. As shown in Figure 3C, optimal degradation of DEHP was observed in the medium devoid of NaCl, achieving 98.4% degradation. However, in the presence of increasing concentration of NaCl, the degradation capability was significantly and concentration-dependently decreased to 42.81, 40, 38.43, 8.07, and 2.44% in the presence of 0.5, 1, 1.5, 2, and 4% NaCl, respectively.

### 3.4. Substrate Spectrum of YC-IL1

The capability of YC-IL1 to degrade other types of PAEs (DMP, DEP, DPrP, DBP, DPeP, DAP, DHP, DHpP, DOP, DNP, DDP, BBP, DCHP, MEHP, and PA) was also tested. As shown in Figure 3D, YC-IL1 metabolized most of the examined PAEs with different efficiencies. In particular, YC-IL1 exhibited excellent capability of 100, 97.0, 96.1, 95.6, 95.4, 93.0, 81.2, and 73.9% toward DMP, DDP, DOP, DEP, DHpP, DHP, BBP, and DNP, respectively. It also showed a moderate degradation capability of 69.0% and 50.7% toward DAP and DCHP, respectively. However, DBP and DPrP were not metabolized by YC-IL1 (Figure 3D). Interestingly, YC-IL1 was capable of using common intermediates of PAE degradation, such as MEHP (80%) and PA (60%), suggesting that this strain could degrade DEHP via MEHP and PA as intermediates.

### 3.5. Degradation Efficiency of DEHP at Maximum and Minimum Concentrations

**Figure 4:**
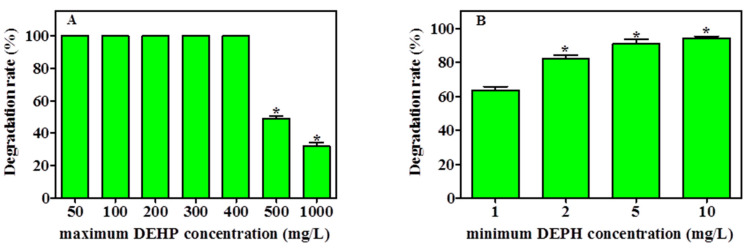
Maximum (**A**) and minimum (**B**) degrading ability of DEHP by YC-IL1 strain after 7 days incubation. Data represent the mean ± SEM of three independent experiments. * indicates the significance difference (*p* < 0.05; one-way ANOVA followed by Dunnett’s test) between the columns denoted with (*) and control; the controls are 100 mg/L for the maximum DEHP concentration (**A**) and 1 mg/L for the minimum DEHP concentration (**B**).

The capability of YC-IL1 to degrade a lower concentration of DEHP and to thrive at higher concentrations was examined. As shown in Figure 4A, YC-IL1 achieved complete degradation of DEHP between 50 and 400 mg/L. However, when the DEHP concentration was increased to 500 and 1000 mg/L, the degradation rate was significantly decreased to 48% and 31.05%, respectively. On the other hand, as shown in Figure 4B, the degradation capability of the lower concentration was directly proportional to the initial concentration used, achieving 63% at 1 mg/L and 94% at 10 mg/L of DEHP after 7 days of cultivation. Hence, these data suggest that YC-IL1 can degrade DEHP at concentrations as low as 1 mg/L and as high as 1000 mg/L.

### 3.6. Deduction of DEHP Degradation Pathway

In order to propose the degradation pathway of DEHP by our strain YC-IL1, intermediate compounds obtained during the degradation were detected by HPLC-QQQ. As shown in Table 1, two intermediates were detected during the incubation period, including MEHP (m/z, 277) with retention time 2.60 min and PA (mz, 164.9&121.0) with retention time 1.443 min.

**Figure 5:**
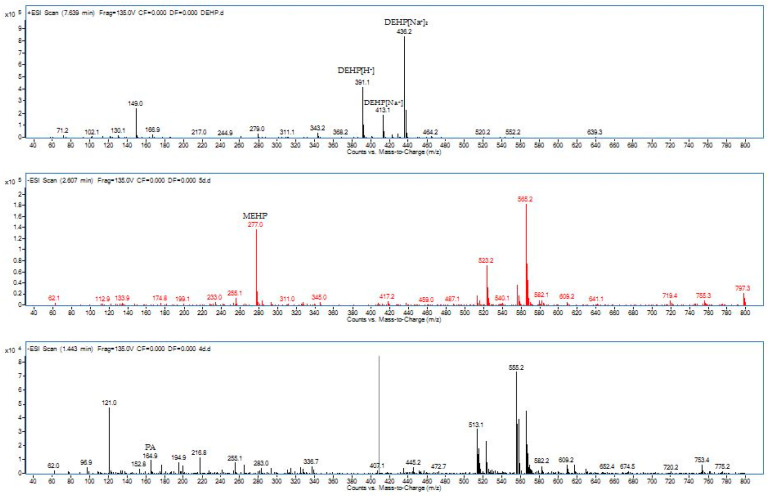
Mass spectra from HPLC-QQQ during DEHP degradation by *Enterobacter* spp. YC-IL1 and detected intermediate compounds, MEHP: mono-ethylhexyl phthalate, PA: phthalate acid.

**Figure 6:**
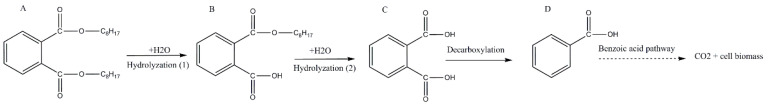
The proposed degradation pathway of di (2-ethylhexyl) phthalate by strain YC-IL1. Letters represent degradation intermediates; (**A**) di (2-ethylhexyl) phthalate, (**B**) monoethylhexyl phthalate, (**C**) phthalic acid, (**D**) benzoic acid.

MEHP started to appear from the fourth day and it was obvious on the fifth day. On the contrary, PA appeared on the fifth day then disappeared, indicating its hydrolysis to benzoic acid (BA) which was not detected because of its low concentration. From the detected intermediates (Figure 5), the degradation pathway was proposed (Figure 6). DEHP was initially hydrolyzed to MEHP at the ester bond. This was further hydrolyzed at the ester bond to phthalic acid. This later was further decarboxylated to BA which was further utilized for cell growth through benzoate metabolic pathway.

### 3.7. Bioremediation of DEHP-Contaminated Soil

**Figure 7:**
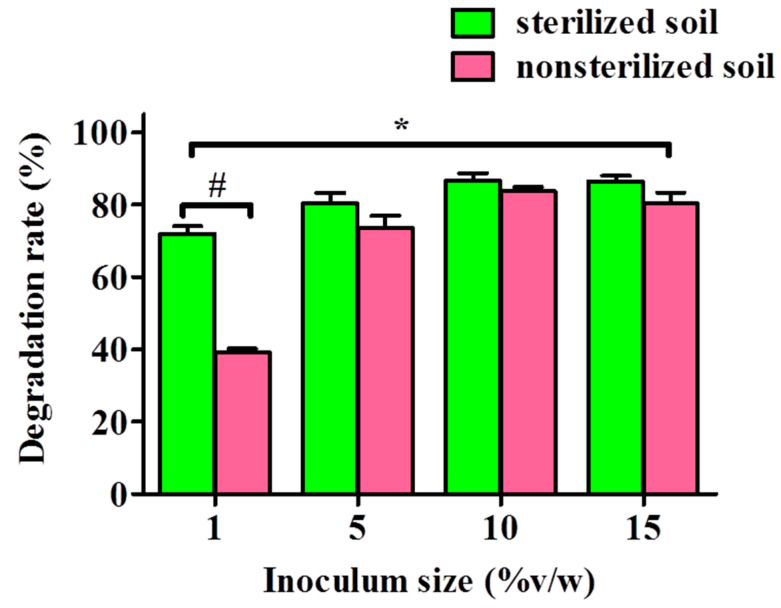
DEHP degradation by strain YC-IL1 with artificial polluted soil. The data represent the mean ± SEM. * *p* < 0.05 two-way ANOVA vs. sterilized soil and ^#^ *p* < 0.05 Bonferroni post-test vs. sterilized soil.

To assess the capability of YC-IL1 to degrade DEHP in the natural environment, garden soil was treated with DEHP at concentration of 100 mg/kg soil and inoculated with different inoculum sizes of YC-IL1 for 6 days of cultivation. As shown in Figure 7, YC-IL-1 degraded DEHP in both sterilized and non-sterilized soil, though the degradation efficiency was significantly greater in the former than the latter, with 72–86.7% and 39.2–86.6%, respectively. Notably, the DEHP degradability was proportional to the inoculum size; meaning it was increased with increasing inoculum size from 1 to 10% but with either no further increase or a slight decrease at 15% inoculum size in both sterilized and non-sterilized soil, respectively (Figure 7).

## 4. Discussion

Increasing concern of PAE toxicity and associated illnesses has resulted in development of numerous methods for their detoxification and elimination. Bioremediation represents an excellent choice due to its superior safety and cost effectiveness. In this scenario, numerous strains have been isolated and implemented for bioremediation purposes [30,31,42]. In the present study, YC-IL1 strain was isolated from Mila province in Algeria and identified as *Enterobacter* spp. YC-IL1 using 16s RNA analysis. This strain was acido- and alkali-tolerant and an efficient degrader of DEHP and other PAE toxicants. To our knowledge, this is the first report describing isolation and characterization of *Enterobacter* spp. strain from Algerian land capable of DEHP degradation.

*Enterobacter* spp. YC-IL1 showed excellent characteristics making it a promising strain that can be implemented in bioremediation of DEHP. First, *Enterobacter* spp. YC-IL1 was capable of degrading DEHP in wide range of pH with optimal activity at 7 and a remarkable activity at alkaline pH (8–9) and acidic pH (3–4), demonstrating both acido- and alkali-tolerance compared with other reported strains [31,39,43]. This characteristic is important because it enables the use of such organism in bioremediation of variable environments. Degradation of DEHP was abolished at pH > 9 probably due to high hydroxyl ion concentration that modifies the structure of the DEHP-degrading enzymes [31]. Second, *Enterobacter* spp. YC-IL1 could degrade DEHP at a wide range of temperatures (25–45 °C) with the optimum efficiency occurring at 30–35 °C. Decrease in degradation rate at higher temperatures (above 40 °C) suggests the death of bacterium and or denaturation of the enzymes involved in DEHP catabolism. Indeed, a too low or too high temperature could hinder the enzyme activity. Few studies have found such strains capable of metabolizing DEHP at a wide range of temperatures [38,39]. Third, *Enterobacter* spp. YC-IL1 withstood and degraded as low as 1 mg/L and as high as 1000 mg/L DEHP. This property of *Enterobacter* spp. YC-IL1 is of great importance because the contaminant level varies from site to site and microbes capable of utilizing low and high concentration of pollutant are of interest for bioremediation [44]. In fact, the concentration of pollutants in the environment is usually very low [45,46]. This situation poses problems for bioremediation because too low pollutant concentration does not ensure the accessibility of pollutants to microbes and fails to induce the expression of functional genes [47]. However, recent studies have demonstrated the presence of PAEs in very high proportions in Chinese soil reaching 58.9 mg/L and even 1232 mg/L in some provinces [48,49], and once this concentration reached or exceeded 100 mg/kg, the microbial biomass and basal respiration in soil were inhibited [50]. Extremely high DEHP concentrations inhibit cell growth and biodegradability [51] which explains the decrease in the degradation efficiency of YC-IL1 when the concentration of DEHP exceeded 400 mg/L. Hence, microorganisms capable of responding and catabolizing low as well as higher concentrations of pollutants are of great interest for bioremediation applications. YC-IL1 showed an excellent capability against low and high concentrations of DEHP compared with other strains, suggesting its applicability in DEHP remediation [52]. Fourth, *Enterobacter* spp. YC-IL1 exhibited a potent degradation capability toward several PAEs other than DEHP such as DMP, DDP, DOP, DEP, DHP and DHPP with efficiencies similar to that of DEHP. This characteristic is also crucial, taking into account the fact that several kinds of PAEs exist simultaneously in DEHP-contaminated environments. Additionally, PAEs which have different branches or ring structure such as DCHP, DEHP and BBP can put steric hindrance to the hydrolase enzymes and degradation by microorganisms [53,54]. Few strains have been reported to display the characteristic of the ability to degrade the most difficult and complex compounds of PAEs such as DCHP, DEHP, and BBP [34,42,55]. For instance, strain *Gordonia* spp. YC-JH1 could degrade BBP, DEHP, and DCHP by 46.9, 25.9, and 10.0%, respectively, in one day. Furthermore, strain *Corynebacterium* spp. DK4 and *Sphigomonas* spp. O18 have a low DCHP degradation efficiency with 24.0% and 32.0% of 100 mg/L DCHP removed after 7 days, respectively. However, the strain YC-IL1 could remove DCHP, DEHP, and BBP by 50.69, 100, and 81.15%, respectively, during the same time period. Of importance, YC-IL1 degraded PAEs with medium and long side chains from DAP to DDP more effectively (68.94–100%) but failed to degrade those with shorter side chains (DPrP and DBP). This finding is in agreement with those reported from *Burkholderia pyrrocinia* B1213 strain [56]. However, it appears controversial to the known fact that PAEs with shorter side chains are more readily degraded by microorganisms than those with longer side chains [51,57,58,59]. Furthermore, YC-IL1 was also able to use the common intermediates of DEHP degradation, such as MEHP and PA, indicating that this strain may degrade DEHP via MEHP and PA as intermediates. Fifth, the *Enterobacter* spp. YC-IL1 could survive in TEM supplemented with 0–4% NaCl, though the DEHP degradation rate was reduced as NaCl concentration increased. High salinity causes plasmolysis and cell lysis due to increase of osmotic potential and affects the metabolic activities of the degrading microbes [60,61]. Nevertheless, salinity tolerance of YC-IL1 strain can be improved with mutagenesis and genetic engineering tools [62]. Altogether, these properties indicate that YC-IL1 is a novel strain capable of bioremediating PAEs pollutants from environments under different conditions.

Generally, the pathway of phthalates in aerobic biodegradation follows two successive stages. First, it starts by biodegradation from phthalic di-esters (PDEs) to phthalic mono-esters (PMEs) and then biodegradation of PMEs to phthalic acid (PA). Secondly, the resulting PA in turn is metabolized to CO_2_ and H_2_O to produce energy [51,63]. During DEHP biodegradation, mono-(2-ehtylhexyl) phthalate (MEHP) as the first metabolite is generated through hydrolysis, followed by the generation of PA after further hydrolysis of MEHP [64]. To explore DEHP degradation pathways by *Enterobacter* spp. YC-IL1, we analyzed the metabolic products by HPLC-QQQ and the intermediates were identified based on the mass-to-charge ratio (m/z). We detected two metabolites: MEHP (retention time 2.607 min) formed by hydrolysis of the ester linkage between each alkyl chain and the aromatic ring of DEHP; and PA (retention time 1.443 min) which appeared later on the fifth day of culture and was lost probably because of its further conversion into benzoic acids. Similar intermediate profiles of DEHP degradation have been reported in different studies including those from our laboratory, therefore we proposed the metabolic pathway for DEHP degradation in Figure 6 [19,30,31,32,39].

Finally, in an attempt to assess the applicability of our novel strain *Enterobacter* spp. YC-IL1 to bioremediating DEHP from the natural environment, we sought to test the capability of YC-IL1 to degrade DEHP in artificially DEHP-contaminated soil. Surprisingly, YC-IL1 biodegraded DEHP from both sterilized and non-sterilized soil with greater efficiency in sterilized soil (Figure 7). The relatively low efficiency of YC-IL1 in non-sterilized soil could be explained by the presence of several microbes (bacteria and fungi) which exercise an inhibitory effect on YC-IL1 to eliminate DEHP in soil. Indeed, soil is rich in diverse microorganisms which are known to produce a plethora of antagonistic compounds such as bacteriocins, antibiotics, and phenolic compounds which may limit the introduced strain from practicing its vital activities normally [65]. Nevertheless, increasing the inoculum size of YC-IL1 to 10% produces a comparable degradability of DEHP between sterilized and non-sterilized soil [66].

## 5. Conclusions

A novel *Enterobacter* spp. YC-IL1 showed an outstanding capability to degrade several PAEs, particularly those with medium and long side chains such as DEHP, DMP, DDP, DOP, DEP, DHpP, and DHP, and PAEs with long chains, branches or ring structures such as BBP, DEHP, and DCHP. YC-IL1 tolerated extreme acidic and alkali pH media such as 3, 4, or 8 and 9 with optimal activity at neutral pH. It also metabolized DEHP in a wide range of moderate temperatures (25–35 °C) and concentrations (1–1000 mg/L). Additionally, this novel strain was also efficient in bioremediating DEHP in artificially contaminated soil. This is the first study to report an acido- and halo-tolerant *Enterobacter* spp. strain capable of degrading a wide spectrum of PAEs, suggesting its applicability in bioremediation of different environmental sites.

## Figures and Tables

**Table 1:** Identification of DEHP biodegradation metabolites.

Chemicals	Formula	MW (g/mol)	Retention Time (min)	m/z	ESI Polarity
DEHP (Substrate)	C_24_H_38_O_4_	390.56	7.369	149.0&391.1&413.1&436.2	Positive
MEHP	C_16_H_22_O_4_	278.34	2.607	277.0	Negative
PA	C_8_H_6_O_4_	166.13	1.443	164.9&121.0	Negative.
